# Molecular characterization of *Coxiella burnetii *isolates by infrequent restriction site-PCR and MLVA typing

**DOI:** 10.1186/1471-2180-6-38

**Published:** 2006-04-26

**Authors:** Nathalie Arricau-Bouvery, Yolande Hauck, Awatef Bejaoui, Dimitrios Frangoulidis, Christelle C Bodier, Armel Souriau, Hermann Meyer, Heinrich Neubauer, Annie Rodolakis, Gilles Vergnaud

**Affiliations:** 1 INRA, Pathologie Infectieuse et Immunologie, 37380 Nouzilly, France; 2 Institute of Genetics and Microbiology, University Paris XI, 91405 Orsay cedex, France; 3 Bundeswehr Institute of Microbiology, Neuherbergstr. 11, 80937 Munich, Germany; 4 Division d'Analyses Biologiques, Centre d'Etudes du Bouchet, BP3, 91710 Vert le Petit, France

## Abstract

**Background:**

*Coxiella burnetii*, the causative agent of Q fever, has a wide host range. Few epidemiological tools are available, and they are often expensive or not easily standardized across laboratories. In this work, *C. burnetii *isolates from livestock and ticks were typed using infrequent restriction site-PCR (IRS-PCR) and multiple loci variable number of tandem repeats (VNTR) analysis (MLVA).

**Results:**

By applying IRS-PCR, 14 *C. burnetii *isolates could be divided into six groups containing up to five different isolates. Clustering as deduced from MLVA typing with 17 markers provided an increased resolution with an excellent agreement to IRS-PCR, and with the plasmid type of each strain. MLVA was then applied to 28 additional *C. burnetii *isolates of different origin and 36 different genotypes were identified among the 42 isolates investigated. The clustering obtained is in agreement with published Multiple Locus Sequence Typing (MLST) data. Two panels of markers are proposed, panel 1 which can be confidently typed on agarose gel at a lower cost and in any laboratory setting (10 minisatellite markers with a repeat unit larger than 9 bp), and panel 2 which comprises 7 microsatellites and provides a higher discriminatory power.

**Conclusion:**

Our analyses demonstrate that MLVA is a powerful and promising molecular typing tool with a high resolution and of low costs. The consistency of the results with independent methods suggests that MLVA can be applied for epidemiological studies. The resulting data can be queried on a dedicated MLVA genotyping Web service.

## Background

Q fever is caused by *Coxiella burnetii*, a small, Gram-negative and strict intracellular bacterium. Although *Coxiella *was historically considered as a member of the genus *Rickettsia*, gene-sequence analysis classified the *Coxiella *genus in the order *Legionellales*, family *Coxiellaceae *with *Rickettsiella *and *Aquicella*, and *C. burnetii *as the only known species of this genus [1]. Q fever is characterized by acute and chronic courses. In humans, acute Q fever usually presents a flu-like, self-limiting disease accompanied by myalgia and severe headache, but complications such as pneumonia or hepatitis may occur. In chronic cases, endocarditis is the main severe complication in patients with valvulopathies. Granulomatous hepatitis, vasculitis, osteomyelitis, post-Q fever fatigue syndrome (QFS) and premature delivery or abortion have also been reported [2,3]. In animals, Q fever affects livestock and is associated with pneumonia and reproductive disorders in livestock, with abortion, stillbirth, delivery of weak and unviable newborns, placentitis, endometritis and infertility [4-6]. *C. burnetii *infections have been reported in a variety of wild and domestic mammals, including dogs, cats and birds. The agent has also been isolated from ticks that are vectors for spreading and maintaining *C. burnetii *in nature [7,8]. The main route of infection is inhalation of contaminated aerosol or dust containing bacteria shed by infected animals with milk, feces, placenta or vaginal secretions [6,9-14]. Oral transmission seems less common, but the consumption of contaminated raw milk and dairy-products represents a potential source of human infection [15].

Human Q fever seems to be re-emerging in various countries as the number of cases described in the literature is increasing. This increase in clinical awareness could result from renewed interest in *Coxiella burnetii *because of bioterrorism concerns since this highly-infectious bacterium is classified as a category B potential biological weapon. However, epidemiological markers are lacking. As a consequence, the source of human infections often remains unidentified but sheep and goats are more frequently involved in the disease cycle than other animal species. In many cases, the occurrence of human cases can be traced back to an infected flock, where the number of aborting ewes has not alerted the farmer [16].

The systematic genotyping of *C. burnetii *isolates would enhance our ability to identify the source of infections and consequently help reduce the number of cases in an outbreak. Although different virulence levels of infections have been observed, it is still not clear whether this is the result of a variability in bacterial virulence factors or whether it depends on the immunological background of the host. Involvement of specific virulence factors, or of particular strains, which can provoke acute or chronic forms, has not yet been demonstrated. Initially, the *com*1 sequence and a certain plasmid profile were assumed to be associated with so-called acute or chronic *C. burnetii *isolates. Recent findings, however, revealed no correlation between these criteria [17-19]. Development of the acute or chronic form of Q fever seems to depend upon the patient's condition and immune status [17,18].

Taking into account the strong similarity or event identity between QpH1 and QpDG, *Coxiella *strains can be divided into four groups based on the occurrence of the plasmids QpH1, QpRS, QpDV and one plasmid (without designation) derived from a chinese *C. burnetii *isolate [20-25]. Plasmidless *C. burnetii *strains carry large plasmid-homologous sequences integrated into the chromosome [26]. Analysis of the genome by techniques such as DNA-DNA hybridization or restriction fragment length polymorphism is hampered, because cultivation of the agent is wearisome. These bacteria are usually grown on cell cultures or embryonated hen's eggs.

Pulsed-field gel electrophoresis has been used for typing of *C. burnetii *strains [27-29], but it is sophisticated and laborious and thus not well suited for routine use. Therefore, the use of newer (usually PCR-based) DNA methods appears to be more appropriate. Infrequent restriction site-PCR (IRS-PCR) has been shown to be a robust method for the molecular characterization of bacteria such as *Bartonella*, *Brucella*, *Legionella*, *Listeria *and *Salmonella *[30-33]. Recently, an MLST (Multiple Loci Sequence Typing) assay was proposed for *C. burnetii *[34]. The assay is based upon the sequencing of 10 short intergenic regions. One hundred and seventy-three isolates of various origins could be separated into 30 different sequence types.

Multiple Loci Variable Number of Tandem Repeats (VNTR) Analysis (MLVA) is a typing method, which is gaining importance due to the availability of whole genome sequences, the often very high discriminatory power, and its very low cost, as compared to MLST for instance. MLVA typing is now considered to be the reference method for many pathogens including *Mycobacterium tuberculosis *[35], *Bacillus anthracis *[36,37], *Yersinia pestis *[38] and is usually applied whenever new genome sequences are released for pathogens of interest [39-43]. In a number of instances, especially in species of recent origin, the discriminatory power of MLVA is much higher than MLST [44]. Freely available resources are accessible over the internet to facilitate the setting-up of new MLVA assays [45,46] or to query existing data [47]. The main aim of the present study is to examine the interest of MLVA to reveal molecular diversity among isolates of *C. burnetii *from livestock and man. A recent investigation lead to the development of a first MLVA assay for *Coxiella burnetii*, using 7 markers and 16 isolates [48]. We explore here additional markers which could be used in an MLVA assay and propose two complementary panel, as recently done for *Brucella *MLVA typing [43]. We compare MLVA to IRS-PCR analysis, and to previous MLST and MLVA reports using published data.

## Results and discussion

### Classification of *C. burnetii *isolates by IRS-PCR

**Figure 1:**
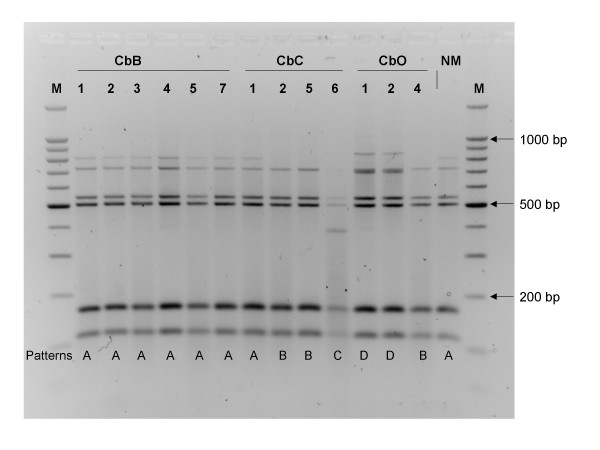
**IRS-PCR pattern of *C. burnetii *isolates using primers PsalT et PS1. **Lane M: molecular weight marker. *C. burnetii *strains and patterns shown are cited in Table 1. NM: Nine Mile reference strain.

Analysis of 14 *C. burnetii *isolates (Table 1) by four different IRS-PCR assays resulted in a total of six patterns (Table 2). The number of DNA fragments generated by IRS-PCR depends on the primers used (i.e. PsalA, PsalC, PsalG, or PsalT), and varied between 6 and 10. The size of the amplicons varied between 100 and 1,000 bp (Figure 1 and data not shown). IRS-PCR assays using PsalG and PS1 generated the highest number of DNA fragments, whereas those using PsalC/PS1 or PsalT/PS1 generated the most diverse patterns. IRS-PCR analysis was made in duplicates and little to no pattern variability between duplicate reactions was found, only minor variations in the intensity of bands. However, the number of DNA fragments was smaller in our study compared to others [31-33], which illustrates the interlaboratory reproducibility problems inherent with multiple loci PCR amplifications.

### MLVA set-up

By analyzing available sequence data, thirty-five tandem repeats with a repeat unit longer than 6 bp, and at least four units were identified in the Microorganism Tandem Repeats Database [46]. One failed to yield a PCR product, 18 were polymorphic and 17 were kept for subsequent analyses (one was not robust enough in our hands, and did not give reproducible results). The 17 markers and corresponding primers are listed in Table 3. The loci are divided in two panels according to repeat unit length. Ten tandem repeats with repeat units equal to or longer that 9 bp which can be confidently typed on agarose gels constitute panel1. This set contains one of the seven loci previously reported by Svraka *et al*. [48], Cox3 (alias ms26). Seven loci have repeat units of 6 or 7 bp, six of which were previously reported [48], and the correspondence is indicated in Table 3. Cox4 (alias ms24) is reported as having a 21 base-pairs repeat unit. However, it is also seen as a 7 bp repeat unit tandem repeat in the tandem repeat database [46], and we observe allele size variations in agreement with this alternative view. Four strains are shared by the two investigations (Nine Mile, Priscilla, Florian, Dugway). Unfortunately, although Svraka *et al*. sequenced all the alleles they observed, the data was not made available [48]. In addition, Svraka *et al*. mention that they observed a discrepancy in the size estimate provided by their capillary electrophoresis equipment compared to the sequence data, and preferred to use the first estimate which is equipment-dependant, and this then makes interlaboratory comparisons more complicated.

A collection of 42 *C. burnetii *isolates could be differentiated by MLVA typing into 22 genotype with panel 1 alone (Figure 3) or 36 genotypes when using the 2 panels (Figure 4). Some isolates have an identical genotype with MLVA. For example, CbB4 and CbB7 are two isolates from French cattle with the same geographic origin. The exact source of the isolates is unknown, it could be from the same herd and explain the identical genotype.

### Genetic relationship of isolates

**Figure 2:**
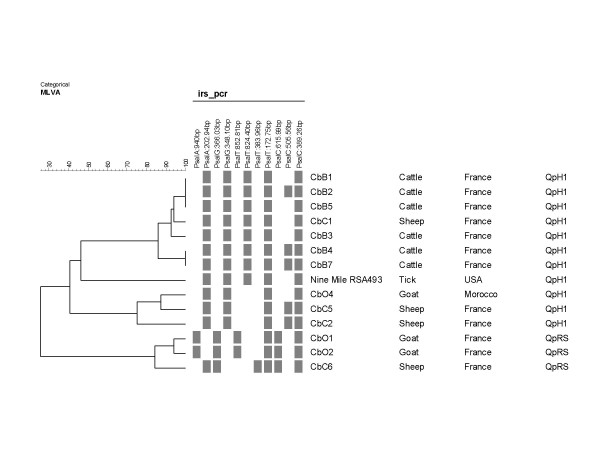
**Comparison of MLVA clustering analysis and IRS-PCR patterns. **Fourteen isolates were analysed with IRS-PCR and MLVA. A schematic view of IRS-PCR data is presented, showing informative bands.

Figure 2 shows the results of MLVA clustering analysis compared to IRS-PCR typing for 14 isolates analyzed with both methods. The two methods are in very good agreement, 6 different genotypes are identified with IRS-PCR as compared to 11 genotypes with MLVA. One discrepancy was observed for strain CbB2. CbB2 is identical to CbB1 and CbB5 by MLVA but shows a different IRS-PCR profile. CbB2 and CbB5 are two isolates obtained in 2001 from neighboring flocks. The affected cows showed different clinical signs (Table 1). CbB2 was isolated from cows having metritis whereas CbB5 had been isolated from cows with abortions. CbB1 originated in placenta of an aborted cow from the same area, but abortion arose before 1998. The two abortive isolates are closely related by the two typing methods.

**Figure 3:**
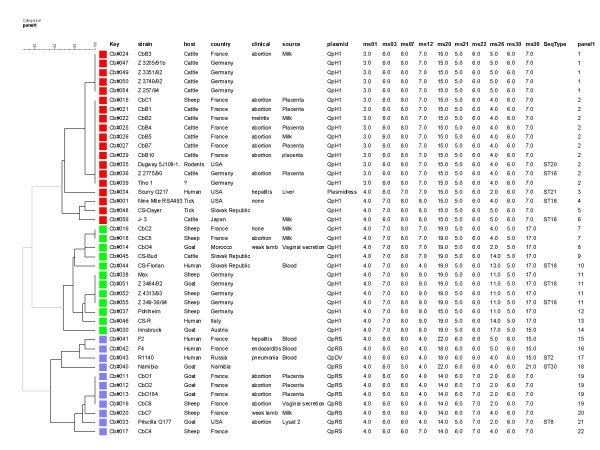
**Dendrogram construct from MLVA Panel 1 data of the42 *C. burnetii *isolates. **Key is a referencing code and refers to a DNA preparation. SeqType, sequence type (ST) as published in [34]. The genotypes have been numbered from 1 to 22 (panel 1 column) for convenience.

**Figure 4:**
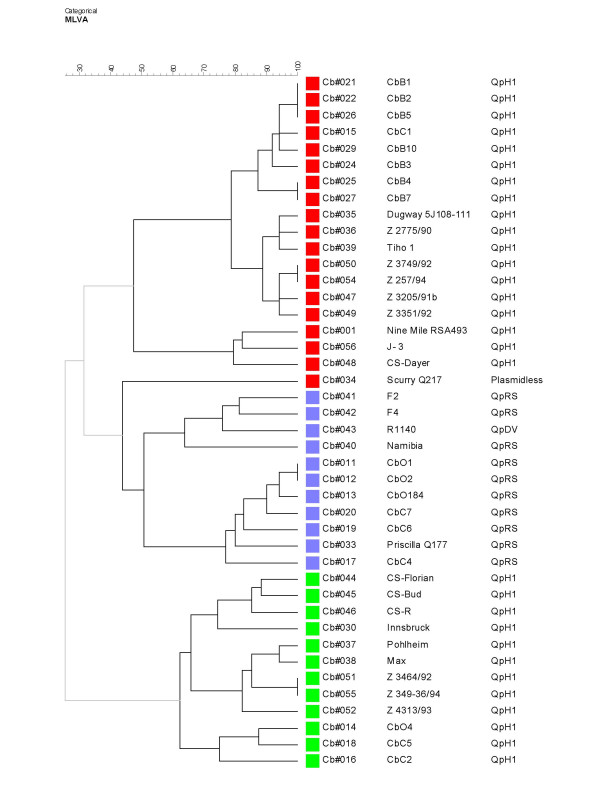
**Dendrogram construct from MLVA Panel 1+2 data ofthe 42 *C. burnetii *isolates. **Thirty-six different genotypes are distinguished. Strains are color-coded with the same code used in Figure 3, to illustrate that the global clustering is preserved when panel 2 is added. The "Scurry" strain is the exception.

Figure 3 shows the result of MLVA clustering from typing 42 *C. burnetii *isolates with panel 1. Twenty-two genotypes are resolved. The Hunter-Gaston diversity index (HGDI) for the panel 1 assay in this collection of strains is 0.92. Three main clusters are identified, comprising respectively 6, 8 and 8 different genotypes. Each cluster contains isolates of various geographic origins. MLVA clustering appears to correctly predict plasmid composition. Eleven reference strains (Scurry Q217, R1140, Namibia, Priscilla Q177, Nine Mile RSA493, J-3, Dugway 5J108-111, Z 2775/90, CS-Florian, Z 3464/92 and Z 349-36/94) were used in our study and the recently published MLST (Multiple Loci Sequence Typing) genotyping assay [34]. In this publication, MLST typing was applied to 173 isolates of various origins. Thirty different sequence types (ST) were reported. The published sequence type (ST) is indicated in Figure 3. The isolates are grouped similarly using both methods, i.e. R1140, Namibia and Priscilla Q177 (ST2, ST30 and ST8) in a first cluster, Nine Mile RSA493, J-3, Dugway 5J108-111 and Z 2775/90 (ST16 and ST20) in a second cluster, and CS-Florian, Z 3464/92 and Z 349-36/94 (ST18) in a third group. Scurry Q217 (ST21) behaves like an outgroup in the MLST investigation, and is similarly poorly connected to the other groups by MLVA (see Figures 3 and 4, the different data sets associate "Scurry" to either the "red" or the "blue" clusters). The two methods are unable to discriminate the German isolates Z 3464/92 and Z 349-36/94. Overall however, and although the methods have not been applied to the same set of isolates, the discriminatory power of MLVA panel 1 alone seems to be comparable to that achieved by the MLST assay, since 22 genotypes are distinguished here among 42 isolates, as compared to the 30 STs observed in 173 various isolates. MLVA distinguishes three ST16 isolates, but does not distinguish the ST20 strain with one of the ST16 strains (at this stage a strain error cannot be excluded). This question would eventually be solved by MLVA typing the 173 isolates previously investigated by MLST.

## Conclusion

Some difficulties of the molecular epidemiology of *C. burnetii *are related to the fastidious growth of this bacterium. MLVA analysis does not require the isolation of the isolates. Genomic analyses of strains can be made directly with DNA purified from milk or placenta. Moreover MLVA typing can be standardized and performed at low cost, thus enabling large-scale molecular epidemiology investigations. Characterizing isolates provoking clearly defined symptoms will allow the identification of strains deserving full genome sequence determination.

Several Q-fever outbreaks have been reported in France but their origin is still unidentified [16]. The lack of epidemiological markers for *C. burnetii *led us to make a global analysis of the available *Coxiella burnetii *genome sequence in order to identify polymorphic tandem repeat loci. Using 17 such loci, we could demonstrate that IRS-PCR can divide 14 *C. burnetii *isolates into 6 different genotypes whereas MLVA differentiates 11 genotypes. An additional limitation of IRS-PCR is that it is essentially a pattern-based assay, which is not easily amenable to interlaboratory standardization and to the making of international databases. MLVA is highly reproducible, has proved to provide efficient discriminatory tools for the molecular typing of bacteria [32], and databases are easy to set-up [45,47] once a few common decisions for allele calling and marker panels have been made [44].

The discriminatory power of MLVA was evaluated using 42 *C. burnetii *isolates. Thirty-six genotypes are identified. Therefore, we recommend MLVA as a valuable tool for epidemiological studies. In particular, we propose to use two panels, panel 1 as a first easy screen, which can be used on agarose gels as well as more sophisticated approaches, and a panel 2, which largely corresponds to the panel previously described by Svraka *et al*. [48] and is best typed using a capillary electrophoresis type of equipment. The present study is an additional step towards the development of MLVA typing for *Coxiella burnetii*. Some of the markers described, in particular panel 2 markers, may eventually turn out to be too variable to be of use (discussed by [44]) when much larger collection of isolates will have been typed. Also, as soon as additional genome sequences will be available, it will be possible to search for additional polymorphic tandem repeats which might have been missed in the present investigation because they have less than 4 repeat units in the Nine Mile RS493 strain genome sequence analyzed here [45].

## Methods

### Bacterial strains and purification

The *C. burnetii *isolates used in this study are listed in Table 1. *Coxiella burnetii *reference strain Nine Mile was provided by AFSSA (Agence Française de Sécurité Sanitaire des Aliments), Sophia Antipolis, France. Isolates were identified as *Coxiella *by phenotypic and genotypic characterization. Isolation of isolates used for IRS-PCR was performed by intraperitoneal inoculation of 3 OF1 mice (8 weeks old) with 0.2 mL of the respective animal samples (Table 1). The mice were killed nine days post inoculation and the spleens were sampled and reinoculated into 6-days-old, specific pathogen free, embryonated hen eggs. The infected yolk sacs (YS) of dead and viable embryos were harvested between 8 and 10 days after inoculation. *C. burnetii *isolates in their 3^rd ^passage in the chicken embryo were aliquoted and frozen at -80°C. Bacterial suspensions were prepared from infected YS by a series of differential sucrose density centrifugations. Prior to the purification process YS were heat inactivated (80°C for 1 hour). This was followed by sonication and by centrifugation for 45 min at 2,000 g in a JOUAN GR412. The supernatant (30 mL) was homogenized with 20 mL of 20% sucrose/phosphate buffer (pH 7.4) and re-centrifuged. After removal of the supernatant, the pellet was suspended in 10 mL Tris-KCl and briefly sonicated again. This bacterial suspension was delicately added up to a centrifuge tube containing 5 mL of 60% sucrose in PBS, 5 mL of 50% sucrose in PBS and 10 mL of 40% sucrose in PBS. Centrifugation was performed at 150,000 *g *for 1 h at 4°C in a Beckman L8-55 ultracentrifuge. *Coxiella *bands were removed, diluted in 30 mL PBS and centrifuged at 150,000 g for 1 hour. The pellet was washed in 5 mL of PBS and centrifuged again.

### DNA preparation

Preparations of purified bacteria were digested with DNAse RQ1 (Promega) at 37°C for 30 min and the reaction was stopped by addition of RQ1 stop solution. This step ensures degradation of cellular DNA. Bacteria were suspended in TNE buffer (50 mM Tris-HCl pH 8.0, 100 mM NaCl, 1 mM EDTA) and digested with proteinase K (Sigma) in the presence of 0.5% sodium dodecyl sulfate at 55°C for 1 h. DNA was extracted with phenol and chloroform, precipitated with ethanol, dried under vacuum, and resuspended in TE buffer (10 mM Tris-HCl pH 8.0, 1 mM EDTA). The DNA concentration and purity was determined by measuring the optical density at both 260 and 280 nm. DNA concentrations were adjusted to 0.1 μg/μL to 0.8 μg/μL. DNAs were stored at -20°C.

### Plasmid specific PCR

The plasmid composition of not previously described isolates was assayed using primers listed in Table 4. PCR amplification conditions are described in Table 4; amplimer lengths were 977 bp for QpH1 and 693 bp for QpRS.

### IRS-PCR

IRS-PCR was performed as described previously [30]. The oligonucleotides that form adapters and are used for PCR amplification are listed in Table 4. The adapters were designed to ligate specifically to the cohesive ends of the *Pst*I and *Sal*I restricted fragments. All oligonucleotides were purchased from Sigma-Genosys. In brief, about 0.5 μg of *Coxiella *DNA was digested with 10 U of *Pst*I (Promega) and 10 U of *Sal*I (Promega) for 2 h 30 min at 37°C in a volume of 15 μL. Ligation of the PstI (20 pmol) and the SalI adapters (20 pmol) was performed by adding 2.5 U of T4 DNA ligase (Promega) in a total volume of 25 μL. The mixture was incubated at 16°C for 2 h and then at 60°C for 20 min to inactivate T4 DNA ligase. The sample was redigested with 5 U of *Pst*I and 5 U *Sal*I at 37°C for 30 min to cleave any restriction sites reformed by ligation, and then was submitted to amplification. Amplification was performed in an iCycler thermocycler (Bio-Rad, Marnes la Coquette, France) with 2.5 μL of template DNA, 0.5 U of *Taq *DNA polymerase (Promega), deoxynucleosides triphosphates (200 μM each) (Promega) and the primers (Ps1 and either PsalA, PsalC, PsalG, or PsalT) in 1× PCR reaction buffer. Amplification consisted of an initial denaturation step at 94°C for 5 min, followed by 30 cycles as indicated in Table 4. All experiments included negative controls that were processed with the samples. The IRS-PCR reaction products were run on 2% (w/v) agarose gels containing 0.5 μg of ethidium bromide per mL.

### Identification of tandem repeats

Methods previously described [36,45,49] and accessible [46] were used to identify tandem repeats in the published genome of *Coxiella burnetii *RS A493 [1].

The various tandem repeat loci are designated by using the nomenclature described previously [35]. For instance Cbu0033-ms01_16bp_5U_198bp (ms01) is a tandem repeat locus at position 33 Kb in the *C. burnetii *RSA493 genome. It has a 16 bp motif and a total PCR product length of 198 bp in the RSA493 strain when using the primers set indicated in Table 3. This allele size is coded as a 5 units allele. The common laboratory name is ms01.

### VNTR amplification and genotyping

PCR amplifications were done in a total volume of 15 μl containing 1 ng of DNA, 1× PCR reaction buffer, 1 U of *Taq *DNA polymerase (Qbiogen, Illkirch, France), 200 μM of each deoxynucleotide triphosphate, and 0.3 μM of each flanking primer (1× PCR buffer is 20 mM Tris pH 8.75, 10 mM KCl, 10 mM (NH4)_2_SO_4_, 2 mM MgSO_4_, 1.5 mM MgCl_2_, 0.1% Triton X-100, 1 M Betaine). Amplifications were performed in a MJ Research PTC200 thermocycler. Initial denaturation at 94°C for 5 minutes was followed by 30 cycles consisting of denaturation at 94°C for 30 s, primer annealing at 60°C for 30 s, and elongation at 70°C for 1 min. The final extension step was at 72°C for 5 min. A different elongation time was used for ms07, ms12 and ms33 (the extension time was 150 seconds at 70°C). Five microliters of amplification product were loaded on a 2% standard agarose gel for panel 1 markers and on a 3% standard agarose gel (Qbiogen, Illkirch, France) for tandem repeats with a 6 or 7 bp repeat unit (panel 2). Gels were stained with ethidium bromide, visualized under UV light, and photographed (Vilber Lourmat, Marnes-la-Vallée, France). The size markers used were a 100-bp or 20-bp ladder (Bio-Rad, Marnes la Coquette, France) according to the tandem repeat unit length. Gel images were managed using the Bionumerics software package (version 4.5, Applied-Maths, Belgium).

### Data analysis

IRS-PCR patterns were analysed using an Alpha Imager Gel Analysis System Fluorchem version 2.00 (Alpha Innotech Corporation) following the manufacturer's recommendations. VNTR alleles size estimates were converted to number of units within a character dataset. Clustering analyses used the categorical coefficient and UPGMA (Unweighted Pair Group Method using Arithmetic averages). The use of the categorical parameter implies that the character states are considered unordered. The same weight is given to a large or a small number of differences in the number of repeats at each locus. Simpson's diversity index was used as suggested by [50].

## Authors' contributions

NAB participated in the design of the study, culture of isolates and molecular genetic studies. YH evaluated tandem repeat markers and carried out all the MLVA molecular genetic studies. GV analyzed the typing data. AB participated in PCR amplification of plasmids. DF and HM selected strains. CCB participated in IRS-PCR molecular genetic studies. AS participated in isolation and culture of isolates, and purification of bacteria. AR participated in search and obtaining grant. NAB, DF, HM and GV drafted the manuscript. All authors read and approved the final manuscript.

## Figures and Tables

**Table 1:** Designation and origin of *Coxiella burnetii *isolates

Strain	Host	Source	Clinical signs	Geographic origin (departement)	Reference
CbB1	Cattle	Placenta	Abortion	France (61)	This study
CbB2	Cattle	Milk	Metritis	France (76)	This study
CbB3	Cattle	Milk	Abortion	France (63)	This study
CbB4	Cattle	Placenta	Abortion	France (61)	This study
CbB5	Cattle	Milk	Abortion	France (76)	This study
CbB7	Cattle	Placenta	Abortion	France (61)	This study
CbB10	Cattle	Placenta	Abortion	France (29)	This study
CbC1	Goat	Placenta	Abortion	France (03)	[51]
CbC2	Goat	Milk	None	France (79)	This study
CbC4	Goat	Milk	-	France (04)	This study
CbC5	Goat	Milk	Abortion	France (82)	This study
CbC6	Goat	Vaginal secretion	Abortion	France (04)	This study
CbC7	Goat	Milk	Weak lamb	France (04)	This study
CbO1	Sheep	Placenta	Abortion	France (37)	This study
CbO2	Sheep	Placenta	Abortion	France (78)	This study
CbO184	Sheep	Placenta	Abortion	France (06)	This study
CbO4	Sheep	Vaginal secretion	Weak lamb	Morocco	This study
Scurry Q217	Human	Liver	Hepatitis	USA	[52]
F2	Human	Blood	Hepatitis	France	[53]
F4	Human	Blood	Endocarditis	France	[53]
R1140	Human	Blood	Pneumonia	Russia	[54]
Namibia	Goat			Namibia	[54]
Priscilla Q177	Goat		Abortion	USA	[52]
Nine Mile RSA493	Tick	Tick	None	USA	[52]
J-3	Cattle	Milk		Japan	[55]
CS-Dayer	Tick			Slovak Republic	[54]
Dugway 5J108-111	Rodents			USA	[52]
Z 2775/90	Cattle	Placenta	Abortion	Germany	[54]
Tiho 1	?			Germany	[54]
Z 3749/92	Cattle			Germany	[54]
Z 257/94	Cattle			Germany	[54]
Z 3205/91b	Cattle			Germany	[54]
Z 3351/92	Cattle			Germany	[54]
CS-Bud	Cattle			Slovak Republic	[54]
CS-R	Human			Italy	[54]
CS-Florian	Human	Blood		Slovak Republic	[54]
Innsbruck	Goat	Placenta	Abortion	Austria	[54]
Z 3464/92	Goat	Placenta	Abortion	Germany	[54]
Z 349-36/94	Sheep	Placenta		Germany	[54]
Max	Sheep	Placenta	Abortion	Germany	[54]
Z 4313/93	Sheep			Germany	[54]
Pohlheim	Sheep			Germany	[54]

**Table 2:** Plasmid types and IRS-PCR patterns of French *Coxiella burnetii *isolates

Isolates	Plasmid type^a^					IRS-PCR pattern
		
		PsalA	PsalC	PsalG	PsalT	Profile type
CbB1	QpH1	A	A	A	A	1
CbB2	QpH1	A	B	A	A	2
CbB3	QpH1	A	A	A	A	1
CbB4	QpH1	A	B	A	A	2
CbB5	QpH1	A	A	A	A	1
CbB7	QpH1	A	B	A	A	2
CbC1	QpH1	A	A	A	A	1
CbC2	QpH1	A	A	A	B	3
CbC5	QpH1	A	B	A	B	4
CbC6	QpRS	A	C	A	C	5
CbO1	QpRS	B	D	B	D	6
CbO2	QpRS	B	D	B	D	6
CbO4	QpH1	A	A	A	B	3
Nine Mile RSA493	QpH1	A	A	A	A	1

^a ^the plasmid type was determined by plasmid-specific PCR

**Table 3:** MLVA loci and properties.

	Locus name	alias^a^	% matches	% G+C content	Primer sequence	Estimated size range (bp)	Unit number^a,b^	No. of alleles^a^	HGDI
Panel 1 markers (minisatellites, repeat unit above 9 base-pairs)	Cbu0033_ms01_16bp_4U_198bp		77	35	L: GGCTCATTCAATTTTAGCTTCG R: AACGTGGGGAAGTTTGTTATTT	182–198	3–4	2	0,47
	Cbu0448_ms03_12bp_7U_229bp		81	18	L: TTGTCGATAAATCGGGAAACTT R: CACTGGGAAAAGGAGAAAAAGA	217–229	6–7	2	0,46
	Cbu0988_ms07_126bp_8U_1112bp		69	6	L: CTCTTAGCCATCGCTTACCACT R: AACGAAAATTGGTTTGCATTTT	734–1112	5–8	4	0,42
	Cbu1316_ms12_126bp_8U_1074bp		74	12	L: GAAAATTGGTTTGCGCTCTG R: CCTTCTCCCAAGAAGTTTAGCC	570–1200	4, 7–9	4	0,67
	Cbu1941_ms20_18bp_15U_402bp		76	6	L: CTGAAACCAGTCTTCCCTCAAC R: CTTTATCTTGGCCTCGCCCTTC	384–528	14–15, 18–19, 22	6	0,7
	Cbu1963_ms21_12bp_6U_210bp		75	42	L: AGCATCTGCCTTCTCAAGTTTC R: TGGGAGGTAGAAGAAAAGATGG	210–222	5–6	2	0,37
	Cbu1980_ms22_11bp_6U_246bp		66	9	L: GGGGTTTGAACATAGCAATACC R: CAATATCTCTTTCTCCCGCATT	246–257	6–7	2	0,28
	Cbu0831_ms26_9bp_4U_127bp	Cox3	94	99	L: AGAATCAAACCTGCAAAACCTT R: TTGATTATTTTGACTTCGCTGGT	109–244	2, 4–5, 11, 13–14, 17 (2,4,13,14,16,18)^c^	6	0,73
	Cbu1351_ms30_18bp_6U_215bp		63	81	L: ATTTCCTCGACATCAACGTCTT R: AGTCGATTTGGAAACGGATAAA	197–215	5–6	2	0,42
	Cbu1941_ms36_9bp_4U_447bp		80	59	L: GAAACCAGTCTTCCCTCAACAG R: ATAACCGTCATCGTCACCTTCT	474–601	7,15,17,21	4	0,55

Panel 2 markers (microsatellites, 6 or 7 bp repeat units)	Cbu0197_ms23_7bp_8U_157bp	Cox6	90	51	L: GGACAAAAATCAATAGCCCGTA R:GAAAACAGAGTTGTGTGGCTTC	122–157	3,5,8 (3,5–6,8)^c^	4	0,67
	Cbu0259_ms24_7bp_27U_344bp	Cox4^d^	94	46	L: ATGAAGAAAGGATGGAGGGACT R: GATAGCCTGGACAGAGGACAGT	204–344	7–13,27 (6,9,12,27)^c^	8	0,79
	Cbu0838_ms27_6bp_4U_276bp	Cox2	90	99	L: TTTTGAGTAAAGGCAACCCAAT R: CAAACGTCGCACTAACTCTACG	264–282	2–5 (2–4)^c^	4	0,73
	Cbu0839_ms28_6bp_6U_276bp	Cox5	94	83	L: TAGCAAAGAAATGTGAGGATCG R: ATTGAGCGAGAGAATCCGAATA	258–288	3–8 (3–7)^c^	6	0,74
	Cbu1418_ms31_7bp_5U_182bp	Cox7	89	41	L: GGGCATCTAATCGAGATAATGG R: TTTGAGAAAATTTTGGGTGCTT	161–182	2–5 (idem)^c^	4	0,51
	Cbu1435_ms33_7bp_9U_262bp		87	50	L: TAGGCAGAGGACAGAGGACAGT R: ATGGATTTAGCCAGCGATAAAA	227–1600	4,6–9	6	0,75
	Cbu1471_ms34_6bp_5U_210bp	Cox1	100	99	L: TGACTATCAGCGACTCGAAGAA R: TCGTGCGTTAGTGTGCTTATCT	192–252	2–5,7–12 (2,3,5,10)^c^	10	0,86

^a ^previously described loci (and corresponding data) is indicated; ^b ^an uninterrupted allele range is indicated by a '-'; ^c ^allele size range reported by [48]; ^d ^Cox4 was initially reported as a 21 bp tandem repeat [48], however we observe a 7 bp repeat unit based variation, in agreement with [46]

**Table 4:** Oligonucleotides used for IRS-PCR and plasmid-specific PCR.

Designation	Sequence	Used for	PCR conditions (°C/seconds)			No. of PCR cycles	Source
					
			Denaturation	Annealing	Extension		
Ps1	5'-GACTCGACTCGCATGCA-3'	Adapter 1, primer IRS-PCR	94/30	55/60 then 60/30	72/90	5 then 30	30
AH2	5'-TGCGAGT-3'	Adapter 1					30
Sal1	5'-PO4-TCGATACTGGCAGACTCT-3'	Adapter 2					30
AX2	5'-GCCAGTA-3'	Adapter 2					30
PsalA	5'-AGAGTCTGCCAGTATCGACA-3'	Primer IRS-PCR	94/30	55/60 then 60/30	72/90	5 then 30	30
PsalC	5'-AGAGTCTGCCAGTATCGACC-3'	Primer IRS-PCR	94/30	55/60 then 60/30	72/90	5 then 30	30
PsalG	5'-AGAGTCTGCCAGTATCGACG-3'	Primer IRS-PCR	94/30	55/60 then 60/30	72/90	5 then 30	30
PsalT	5'-AGAGTCTGCCAGTATCGACT-3'	Primer IRS-PCR	94/30	55/60 then 60/30	72/90	5 then 30	30
CB5	5'-ATAATGAGATTAGAACAACCAAGA-3'	Primer QpH1 PCR	94/120	53/60	72/120	35	56
CB6	5'-TCTTTCTTGTTCATTTTCTGAGTC-3'	Primer QpH1 PCR	94/120	53/60	72/120	35	56
QpRS1	5'-CTCGTACCCAAAGACTATGAATATATCC-3'	Primer QpRS PCR	94/60	54/60	72/120	36	56
QpRS2	5'-AACACCGATCAATGCGACTAGCCC-3'	Primer QpRS PCR	94/60	54/60	72/120	36	56

## References

[B1] Seshadri R, Paulsen IT, Eisen JA, TD R, Nelson KE, Nelson WC, Ward NL, Tettelin H, Davidsen TM, Beanan MJ, Deboy RT, Daugherty SC, Brinkac LM, Madupu R, Dodson RJ, Khouri HM, Lee KH, Carty HA, Scanlan D, Heinzen RA, Thompson HA, Samuel JE, Fraser CM, Heidelberg JF (2003). Complete genome sequence of the Q-fever pathogen *Coxiella burnetii*. Proc Natl Acad Sci USA.

[B2] Hatchette TF, Hayes M, Merry H, Schlech WF, Marrie TJ (2003). The effect of *C. burnetii *infection on the quality of life of patients following an outbreak of Q fever. Epidemiol Infect.

[B3] Raoult D, Tissot-Dupont H, Foucault C, Gouvernet J, Fournier PE, Bernit E, Stein A, Nesri M, Harle JR, Weiller PJ (2000). Q fever 1985–1998. Clinical and epidemiologic features of 1,383 infections. Medicine.

[B4] Bildfell RJ, Thomson GW, Haines DM, McEwen BJ, Smart N (2000). *Coxiella burnetii *infection is associated with placentitis in cases of bovine abortion. J Vet Diagn Invest.

[B5] Moeller RB (2001). Causes of caprine abortion: diagnostic assessment of 211 cases (1991–1998). J Vet Diagn Invest.

[B6] To H, Htwe KK, Yamasaki N, Zhang GQ, Ogawa M, Yamaguchi T, Fukushi H, Hirai K (1995). Isolation of *Coxiella burnetii *from dairy cattle and ticks, and some characterization of the isolates in Japan. Microbiol Immunol.

[B7] Babudieri B (1959). Q fever: a zoonosis. Adv Vet Sci.

[B8] Lang GH (1990). Coxiellosis (Q fever) in animals.

[B9] Martinov SP, Neikov P, Popov GV (1989). Experimental Q fever in sheep. Eur J Epidemiol.

[B10] Palmer NC, Kierstead M, Key DW, Williams JC, Peacock MG, Vellend H (1983). Placentitis and abortion in goats and sheep in Ontario causedby *Coxiella burnetii*. Can Vet J.

[B11] Stein A, Raoult D (1999). Pigeon pneumonia in provence: a bird-borne Q fever outbreak. Clin Infect Dis.

[B12] Stoker MGP, Brown RD, Kett FJL, Collings PC, Marmion BP (1955). Q fever in Britain. Isolation of *Rickettsia burneti *from placenta and wool of sheep in an endemic area. J Hyg.

[B13] Tissot-Dupont H, Torres S, Nezri M, Raoult D (1999). Hyperendemic focus of Q fever related to sheep and wind. Am J Epidemiol.

[B14] Welsh HH, Lennette EH, Abinanti FR, Winn JF (1957). Airborne transmission of Q fever: the role of parturition in generation of infective aerosol. Ann New York Acad SCI.

[B15] Fishbein DB, Raoult D (1992). A cluster of *Coxiella burnetii *infections associated with exposure to vaccinated goats and their unpasteurized dairy products. Am J Trop Med Hyg.

[B16] Berri M, Rousset E, Champion JL, Arricau-Bouvery N, Russo P, Pepin M, Rodolakis A (2003). Ovine manure used as a garden fertiliser is suspected to be a contamination source of two human Q fever cases. Vet record.

[B17] Stein A, Raoult D (1993). Lack of pathotype specific gene inhuman *Coxiella burnetii *isolates. Microb Pathog.

[B18] Thiele D, Willems H (1994). Is plasmid based differentiation of *Coxiella burnetii *in 'acute' and 'chronic' isolates still valid?. Eur J Epidemiol.

[B19] Zhang GQ, To H, Yamaguchi T, Fukushi H, Hirai K (1997). Differentiation of *Coxiella burnetii *by sequence analysis of the gene (com1) encoding a 27-kDa outer membrane protein. Microbiol Immunol.

[B20] Hendrix LR, Samuel JE, Mallavia LP (1991). Differentiation of *Coxiella burnetii *isolates by analysis of restriction-endonuclease-digested DNA separated by SDS-PAGE. J Gen Microbiol.

[B21] Lautenschlager S, Willems H, Jäger C, Baljer G (2000). Sequencing and characterization of the cryptic plasmid QpRS from *Coxiella burnetii*. Plasmid.

[B22] Ning Z, Yu SR, Quan YG, Xue Z (1992). Molecular characterization of cloned variants of *Coxiella burnetii *isolated in China. Acta Virol.

[B23] Thiele D, Willems H, Haas M, Krauss H (1994). Analysis of the entire nucleotide sequence of the cryptic plasmid QpH1 from Coxiella burnetti. Eur J Epidemiol.

[B24] Valkova D, Kazar J (1995). A new plasmid (QpDV) common to *Coxiella burnetii *isolates associated with acute and chronic Q fever. FEMS Microbiol Lett.

[B25] Jäger C, Lautenschlager S, Willems H, Baljer G (2002). *Coxiella burnetii *plasmid types QpDG and QpH1 are closely related and likely identical. Vet Microbiol.

[B26] Willems H, Ritter M, Jäger C, Thiele D (1997). Plasmid-homologous sequences in the chromosome of plasmidless *Coxiella burnetii *Scurry Q217. J Bacteriol.

[B27] Heinzen R, Stiegler GL, Whiting LL, Schmitt SA, Malavia LP, Frazier ME (1990). Use of pulse field gel electrophoresis to differentiate *Coxiella burnetii *strains. Ann NY Acad Sci.

[B28] Jäger C, Willems H, Thiele D, Baljer G (1998). Molecular characterization of *Coxiella burnetii *isolates. Epidmiol Infect.

[B29] Thiele D, Willems H, Kopf G, Krauss H (1993). Polymorphism in DNA restriction patterns of *Coxiella burnetii *isolates investigated by pulse field gel electrophoresis and image analysis. Eur J Epidemiol.

[B30] Cloeckaert A, Grayon M, Grepinet O, Boumedine KS (2003). Classification of *Brucella *strains isolated from marine mammals by infrequent restriction site-PCR and development of specific PCR identification tests. Microbes Infect.

[B31] Franciosa G, Tartaro S, Wedell-Neergaard C, Aureli P (2001). Characterization of *Listeria monocytogenes *strains involved in invasive and noninvasive listeriosis outbreaks by PCR-based fingerprinting techniques. Appl Environ Microbiol.

[B32] Garaizar J, Lopez-Molina N, Laconcha I, Lau Baggesen D, Rementeria A, Vivanco A, Audicana A, Perales I (2000). Suitability of PCR fingerprinting, infrequent-restriction-site PCR, and pulsed-field gel electrophoresis, combined with computerized gel analysis, in library typing of *Salmonella enterica *serovar Enteritidis. Appl Environ Microbiol.

[B33] Handley SA, Regnery RL (2000). Differentiation of pathogenic *Bartonella *species by infrequent restriction site PCR. J Clin Microbiol.

[B34] Glazunova O, Roux V, Freylikman O, Sekeyova Z, Fournous G, Tyczka J, Tokarevich N, Kovacava E, Marrie TJ, Raoult D (2005). *Coxiella burnetii *genotyping. Emerg Infect Dis.

[B35] Le Flèche P, Fabre M, Denoeud F, Koeck JL, Vergnaud G (2002). High resolution, on-line identification of strains from the *Mycobacterium tuberculosis *complex based on tandem repeat typing. BMC Microbiol.

[B36] Le Flèche P, Hauck Y, Onteniente L, Prieur A, Denoeud F, Ramisse V, Sylvestre P, Benson G, Ramisse F, Vergnaud G (2001). A tandem repeats database for bacterial genomes: application to the genotyping of *Yersinia pestis *and *Bacillus anthracis*. BMC Microbiol.

[B37] Lista L, Faggioni G, Valjevac S, Ciammaruconi A, Vaissaire J, le Doujet C, Gorge O, De Santis R, Carattoli A, Ciervo A, Fasanella A, Orsini F, D'Amelio R, Pourcel C, Cassone A, Vergnaud G (2006). Genotyping of *Bacillus anthracis *strains based on automated capillary 25-loci Multiple Locus Variable-Number Tandem Repeats Analysis. BMC Microbiology.

[B38] Pourcel C, Andre-Mazeaud F, Neubauer H, Ramisse F, Vergnaud G (2004). Tandem repeats analysis for the high resolution phylogenetic analysis of *Yersinia pestis*. BMC Microbiol.

[B39] Pourcel C, Vidgop Y, Ramisse F, Vergnaud G, Tram C (2003). Characterization of a Tandem Repeat Polymorphism in *Legionella pneumophila *and Its Use for Genotyping. J Clin Microbiol.

[B40] Onteniente L, Brisse S, Tassios PT, Vergnaud G (2003). Evaluation of the polymorphisms associated with tandem repeats for *Pseudomonas aeruginosa *strain typing. J Clin Microbiol.

[B41] Ramisse V, Houssu P, Hernandez E, Denoeud F, Hilaire V, Lisanti O, Ramisse F, Cavallo JD, Vergnaud G (2004). Variable number of tandemrepeats in *Salmonella enterica *subsp. *enterica *fortyping purposes. J Clin Microbiol.

[B42] Koeck JL, Njanpop-Lafourcade BM, Cade S, Varon E, Sangare L, Valjevac S, Vergnaud G, Pourcel C (2005). Evaluation and selection of tandem repeat loci for *Streptococcus pneumoniae *MLVA strain typing. BMC Microbiol.

[B43] Le Fleche P, Jacques I, Grayon M, Al Dahouk S, Bouchon P, Denoeud F, Nockler K, Neubauer H, Guilloteau LA, Vergnaud G (2006). Evaluation and selection of tandem repeat loci for a *Brucella *MLVA typing assay. BMC Microbiol.

[B44] Vergnaud G, Pourcel C, Stackebrandt E (2006). Multiple Locus VNTR (Variable Number of Tandem Repeat) Analysis (MLVA). Molecular Identification, Systematics and Population Structure of Prokaryotes.

[B45] Denoeud F, Vergnaud G (2004). Identification of polymorphic tandem repeats by direct comparison of genome sequence from different bacterial strains: a Web-based ressource. BMC Bioinformatics.

[B46] The Microorganisms Tandem Repeats Database. http://minisatellites.u-psud.fr.

[B47] The MLVA Web Service. http://bacterial-genotyping.igmors.u-psud.fr.

[B48] Svraka S, Toman R, Skultety L, Slaba K, Homan WL (2006). Establishment of a genotyping scheme for *Coxiella burnetii*. FEMS Microbiol Lett.

[B49] Vergnaud G, Denoeud F (2000). Minisatellites: Mutability and Genome Architecture. Genome Res.

[B50] Hunter PR, Gaston MA (1988). Numerical index of the discriminatory ability of typing systems: an application of Simpson's index of diversity. J Clin Microbiol.

[B51] Arricau Bouvery N, Souriau A, Lechopier P, Rodolakis A (2003). Experimental *Coxiella burnetii *infection in pregnant goats: excretion routes. Vet Res.

[B52] Samuel JE, Frazier ME, Mallavia LP (1985). Correlation of plasmid type and disease caused by *Coxiella burnetii*. Infect Immun.

[B53] Stein A, Raoult D (1992). Detection of *Coxiella burnetti *by DNA amplification using polymerase chain reaction. J Clin Microbiol.

[B54] Willems H, Thiele D, Krauss H (1993). Plasmid based differentiation and detection of *Coxiella burnetii *in clinical samples. Eur J Epidemiol.

[B55] Nguyen SV, Hirai K (1999). Differentiation of *Coxiella burnetii *isolates by sequence determination and PCR-restriction fragment length polymorphism analysis of isocitrate dehydrogenase gene. FEMS Microbiol Lett.

[B56] Zhang GQ, Hotta A, Mizutani M, Ho T, Yamaguchi T, Fukushi H, Hirai K (1998). Direct identification of *Coxiella burnetii *plasmids in human sera by nested PCR. J Clin Microbiol.

